# Preparation and efficacy of antibacterial methacrylate monomer-based polymethyl methacrylate bone cement containing N-halamine compounds

**DOI:** 10.3389/fbioe.2024.1414005

**Published:** 2024-05-28

**Authors:** Rui Guo, Yu-Chen Kan, Yang Xu, Lu-Yang Han, Wen-Han Bu, Long-Xu Han, Yin-Yu Qi, Jian-Jun Chu

**Affiliations:** ^1^ Department of Orthopedics, The Second People’s Hospital of Hefei, Hefei Hospital Affiliated to Anhui Medical University, Hefei, Anhui, China; ^2^ The Fifth Clinical Medical School of Anhui Medical University, Hefei, Anhui, China; ^3^ School of Food and Biological Engineering, Hefei University of Technology, Hefei, Anhui, China

**Keywords:** antibacterial cement, bone cement modification, infection, N-halamine compound, polymethyl methacrylate, *Staphylococcus aureus*

## Abstract

**Introduction:**

Our objective in this study was to prepare a novel type of polymethyl methacrylate (PMMA) bone cement, analyze its material properties, and evaluate its safety and antibacterial efficacy.

**Methods:**

A halamine compound methacrylate antibacterial PMMA bone cement containing an N-Cl bond structure was formulated, and its material characterization was determined with Fourier transform infrared spectroscopy (FT-IR) and ^1^H-NMR. The antibacterial properties of the material were studied using contact bacteriostasis and releasing-type bacteriostasis experiments. Finally, *in vitro* and *in vivo* biocompatibility experiments were performed to analyze the toxic effects of the material on mice and embryonic osteoblast precursor cells (MC3T3-E1).

**Results:**

Incorporation of the antibacterial methacrylate monomer with the N-halamine compound in the new antibacterial PMMA bone cement significantly increased its contact and releasing-type bacteriostatic performance against *Staphylococcus aureus*. Notably, at 20% and 25% additions of N-halamine compound, the contact and releasing-type bacteriostasis rates of bone cement samples reached 100% (*p* < 0.001). Furthermore, the new antibacterial bone cement containing 5%, 10%, and 15% N-halamine compounds showed good biocompatibility *in vitro* and *in vivo*.

**Conclusion:**

In this study, we found that the novel antibacterial PMMA bone cement with N-halamine compound methacrylate demonstrated good contact and releasing-type bacteriostatic properties against *S. aureus*. In particular, bone cement containing a 15% N-halamine monomer exhibited strong antibacterial properties and good *in vitro* and *in vivo* biocompatibility.

## 1 Introduction

Antibiotic-loaded bone cement (ALBC) has been extensively used in the clinical treatment of periprosthetic joint infection (PJI). Common ALBCs incorporate vancomycin, gentamicin, and clindamycin into the bone cement. (Nau et al., 2016; Boelch et al., 2017; Balato et al., 2019). While research on the physical doping strategies of antibiotic bone cement has intensified, its clinical application has also exposed several of its limitations. First, mere physical doping of antibiotics into the bone cement leads to the uneven distribution of antibiotics in the bone cement, thereby hindering effective antibiotic release. Consequently, there is an initial burst of antibiotic release on the surface of polymethyl methacrylate (PMMA) bone cement (the release peak often occurs within two to 3 days). The antibiotic release concentration subsequently decreases, and the prolonged local low-dose release of antibiotics can result in catastrophic bacterial resistance problems. (Schnieders et al., 2011; Al Thaher et al., 2017; Miao et al., 2023). Therefore, there is an urgent need to develop a new antibacterial strategy for bone cement to address the challenges associated with bacterial resistance in traditional ALBC formulations.

In non-leaching bone cement (NLBC), the antibacterial groups are mainly attached to PMMA through covalent linkage, thereby exerting antibacterial effects by destroying the cell membrane of bacteria adhering to the bone cement surface. Consequently, NLBC is not prone to bacterial resistance while effectively inhibiting bacterial growth through contact bacteriostatic mechanisms. (Vasilev et al., 2009; Jiao et al., 2017; Ghimire and Song, 2021; Bhattacharjee et al., 2022). NLBCs containing heterocyclic compounds and quaternary ammonium salts have been the most extensively studied. (Deb et al., 2008; Chu et al., 2022). N-halamine compounds have garnered significant attention in research and application due to their antibacterial mechanisms, such as contact and releasing-type bacteriostasis, as well as their favorable biocompatibility. (Luo et al., 2011; Bai et al., 2016; Liu et al., 2018; Ma W. et al., 2019; Ma Y. et al., 2019). However, so far, orthopedic implant materials have remained understudied in this context, highlighting the potential for research and development of antibacterial PMMA bone cement containing methacrylate monomer with halamine compounds. This is expected to open up new prospects for clinical applications in the prevention and treatment of orthopedic implant infections.

In this study, we designed and synthesized a new antibacterial methacrylate monomer of an N-halamine compound containing an N-Cl bond structure. Subsequently, we formulated a new type of antibacterial PMMA bone cement incorporating this N-halamine compound and analyzed its material characterization, antibacterial, and biocompatibility properties so as to develop a safe and efficient new antibacterial bone cement.

## 2 Materials and methods

### 2.1 Materials

The following materials were used in this study: 4-Methylpiperidinol, methacrylic acid (MMA), sodium hypochlorite (NaClO), dichloromethane (DCM), 1-(3-Dimethylaminopropyl)-3-ethylcarbodiimide hydrochloride (EDCI), 4-dimethylaminopyridine (DMAP) (Shanghai Macleane Biochemical Co., Ltd.); Polymethyl methacrylate (PMMA) (Sigma-Aldrich, USA; 120,000 mesh), benzoyl peroxide (BPO), barium sulfate (BaSO_4_), N,N-dimethyl-p-toluidine (DMPT) (Shanghai Macleane Biochemical Co., Ltd.); Fourier transform infrared spectroscopy (FT-IR), Nuclear Magnetic Resonance Spectrometer (Bruker, Germany); *Staphylococcus aureus* strain (ACTT 25923) (Hefei Hospital, Anhui Medical University); and C57 mice (SPF (Beijing) Biotechnology Co., Ltd.).

This study was approved by the Biomedical Ethics Committee of the institution.

### 2.2 Methods

#### 2.2.1 Synthesis of the antibacterial methacrylate monomer with an N-halamine compound containing an N-Cl bond structure

**FIGURE 1:**
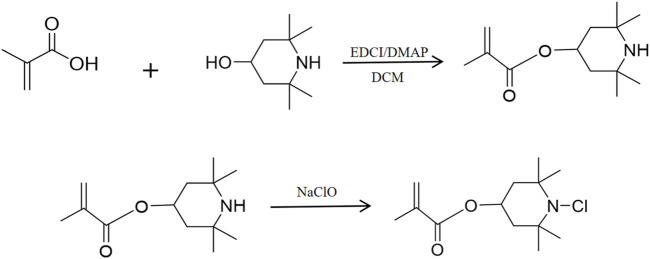
Synthesis of a methacrylate antimicrobial monomer with halamine containing an N-Cl bond structure.

The synthesis path of the antibacterial methacrylate monomer with an N-halamine compound containing an N-Cl bond structure is shown in Figure 1. Initially, 50 mL of dichloromethane (DCM) was added to the round-bottom flask, then 3.15 g (20 mmol) of 4-methylpiperidinol and a slight excess of methacrylic acid (MMA) (24 mmol) were added and stirred until the raw materials were completely dissolved. After this, 1-(3-Dimethylaminopropyl)-3-ethylcarbodiimide hydrochloride (EDCI)/4-dimethylaminopyridine (DMAP) (4 mmol) was added as the reaction catalyst and stirring was continued at room temperature for 24 h. When the reaction was complete, the product was obtained via extraction, concentration, and purification processes. The product was mixed with excess sodium hypochlorite (30 mmol), 4 mL of distilled water, and 16 mL of tert-butanol and stirred at room temperature for 24 h. The final product obtained after decompression was a light yellow and transparent viscous liquid with a yield of 80%.

#### 2.2.2 Fourier transform infrared spectroscopy (FT-IR) analysis of the novel antibacterial bone cement

The samples to be tested were prepared in the form of granules by mixing PMMA bone cement and bone cement with varying concentrations (5%, 15%, and 25%) of the N-halamine compound monomer with potassium bromide. Functional group analysis of novel antibacterial cement composites containing different concentrations of the N-halamine compound was performed using FT-IR spectroscopy and recorded on a spectrometer (BRUKER VECTOR-22) with a frequency range of 4,000–400 cm^−1^.

#### 2.2.3 ^1^H-NMR analysis of the methacrylate monomer containing the N-halamine compound

Deuterated chloroform (CDCl_3_) was used as the solvent, and a small amount of methacrylate monomer containing the N-halamine compound was added to CDCl_3_ to ensure complete dissolution. Tetramethylsilane was added as an internal standard, and ^1^H-NMR determination was performed using a nuclear magnetic resonance spectrometer (BRUKER-400 MHz).

#### 2.2.4 Preparation of antibacterial PMMA bone cement samples with the halamine compound containing the N-Cl bond structure

PMMA particles were ground into a powder with a mesh size of 160–200, mixed with BPO and barium sulfate to obtain the solid-phase powder of the bone cement. The liquid-phase preparation was a mixture of the N-halamine compound antimicrobial monomer and MMA. The solid and liquid phases were mixed in specific ratios as detailed in Table 1 and poured into molds to create cylindrical samples with a length of (12.0 ± 0.1) mm and a diameter of (6.0 ± 0.1) mm. This process yielded six distinct groups: a blank control group (PMMA group) and bone cement formulation groups containing 5%, 10%, 15%, 20%, and 25% N-halamine compound monomers, respectively.

**TABLE 1:** Composition of the antibacterial PMMA bone cement with halamine compounds containing N-Cl bond structures (based on a total mass of 1 g).

Formulation	Powder(mg)			Liquid(mg)	
	PMMA	BaSO_4_	BPO	MMA/N-Cl	DMPT
Plain cement	525	100	13	355/0	7
5% N-Cl	525	100	13	337.25/17.75	7
10% N-Cl	525	100	13	319.5/35.5	7
15% N-Cl	525	100	13	301.75/53.25	7
20% N-Cl	525	100	13	284/71	7
25% N-Cl	525	100	13	266.25/88.75	7

#### 2.2.5 Preparation and grouping of sample extracts

The bone cement samples, that is, cylindrical specimens with a length of (12.0 ± 0.1) mm and a diameter of (6.0 ± 0.1) mm, were immersed in cell culture medium or normal saline (0.2 g/mL) and subsequently incubated at 37°C and 5% CO_2_ for 24 h. The extract was obtained and stored in a 4°C freezer for later use, with the pH value adjusted to 7.4. (Li, 2014). Additionally, untreated cell culture medium and normal saline were used as the blank control group. The extracts obtained from the cell culture medium and normal saline were intended for use in cell proliferation and toxicity experiments and acute toxicity tests, respectively.

#### 2.2.6 Determination of the antibacterial properties of the new antibacterial bone cement

Using aseptic procedures, *S. aureus* cultures were retrieved and passaged to obtain a bacterial solution with a concentration of 0.5 × 10^8^ CFU/mL. The six groups of bone cement samples, that is, cylindrical specimens with a length of (12.0 ± 0.1) mm and a diameter of (6.0 ± 0.1) mm, were soaked in glass test tubes filled with 1 mL of bacterial solution and incubated at 37°C for 6 h. The samples were removed (the residual bacterial solution was retained for later use), rinsed with normal saline, and subjected to ultrasonic vibrations for 3 min. A 40-μL aliquot of the vibrated liquid was diluted and plated. After culture for 24 h, this was followed by colony counting to calculate the contact bacteriostasis rate. Similarly, the residual bacterial solution was diluted and plate-coated, counted after culture for 24 h, and the releasing-type bacteriostasis rate was calculated. A total of five samples were tested in each group. The antibacterial rate was determined using the formula:

$$Antibacterial\;rate=\frac{A-B}{A}\times 100\%$$

In the above formula, A represents the number of colonies in the blank bone cement group, and B represents the number of colonies in each experimental group.

#### 2.2.7 Determination of biocompatibility of the new antibacterial bone cement

##### 2.2.7.1 *In vitro* cell proliferation and toxicity experiments

Following aseptic procedures, mouse embryonic osteoblast precursor cells (MC3T3-E1) were isolated and suspended in 37°C double-distilled water. After centrifugation, the cells were resuspended in medium and cultured in flasks at 37°C with 5% CO_2_. Passage was initiated when the cell confluence reached 80%. The cells were dissociated into single-cell suspensions and seeded in 96-well plates, followed by culturing under the same conditions for 24 h. Subsequently, the original medium was replaced with cell culture medium extracts from each group of samples and co-cultured with MC3T3-E1 cells. Cell Counting Kit-8 (CCK-8) was used to detect cell proliferation on days 1, 3, and 5 of cell culture. The optical density (OD) was measured using an enzyme-linked immunoassay monitor at a wavelength of 450 nm, and the relative growth rate (RGR) of cells in each group was calculated. Finally, the morphological changes and cytotoxicity grades of cells were evaluated based on the criteria outlined in GB/T 16886. (State Administration for Market Regulation of China, 2022). The following formula was used to calculate the RGR:

$$RGR=\frac{{OD}_{T}-{OD}_{R}}{{OD}_{N}-{OD}_{R}}\times 100\%$$

In the above formula, *OD*_*T*_ refers to the absorbance of the experimental group, *OD*_*N*_ refers to the absorbance of the blank control group, and *OD*_*R*_ refers to the absorbance of cell-free medium.

##### 2.2.7.2 Acute toxicity test in mice

A total of 35 C57 mice weighing 16–21 g were randomly divided into seven groups, with five mice in each group. Prior to experimentation, the body weights of all mice were measured and recorded. Mice in each group were intraperitoneally injected with normal saline, PMMA, and normal saline extracts containing 5%, 10%, 15%, 20%, and 25% N-halamine compound monomers, respectively, with an injection volume of 50 mL/kg per mouse. The injection site was located 0.5 cm below the midline of the lower abdomen in mice, where the needle was inserted into the abdominal cavity at a 30° angle to the skin surface and the extract was administered. All mice were raised in the laboratory animal room of Anhui Medical University, maintained in a ventilated and dry environment of 18°C–22°C. Observations regarding the general state, changes in body weight, and any signs of toxic reactions in the mice were conducted at 24, 48, and 72 h after the injection. The mice were euthanized after 72 h, and their livers and kidneys were harvested for hematoxylin-eosin staining. To minimize the pain, fear, and stress experienced by the experimental animals as much as possible, all mice were euthanized by cervical dislocation. This procedure involves firmly grasping the tail of mice with the right hand and pulling it backward, while simultaneously applying downward pressure on the head with the thumb and index finger of the left hand, thereby severing the spinal cord from the brain stem, resulting in the immediate death of the mice. Subsequently, a histological examination of the tissues was performed under a microscope. The assessment of acute systemic toxicity was carried out according to the guidelines outlined in GB/T 16886. (State Administration for Market Regulation of China, 2022).

#### 2.2.8 Statistical analysis

SPSS 22.0 was used for statistical analysis. Measurement data were expressed as $\bar{x}$ ±s. The *t*-test was used for comparison between groups, and the repeated measures analysis of variance (ANOVA) was used for comparing data between two groups at different time points. Counting data was expressed as percentages, and the chi-square test was used for comparison between groups. The difference was considered statistically significant at a threshold of *p* < 0.05.

## 3 Results

### 3.1 Fourier transform infrared spectroscopy (FT-IR) analysis of the novel antibacterial bone cement

**FIGURE 2:**
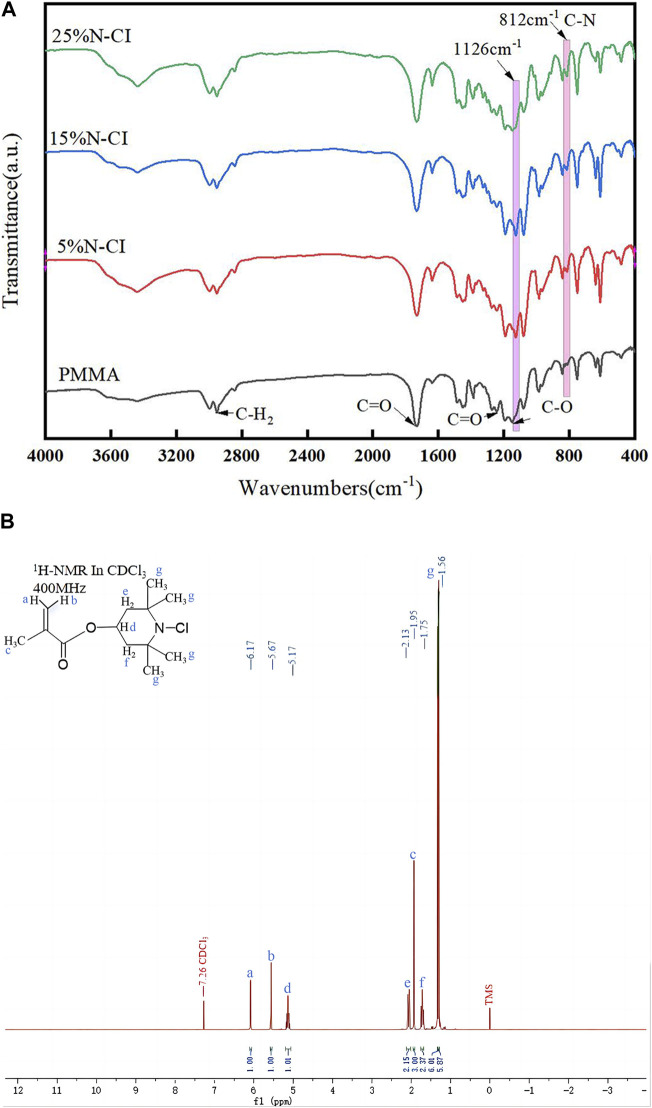
(Continued). **(A)** Infrared spectra of the PMMA bone cement and bone cements with 5%, 15%, and 25% N-halamine compound monomers. **(B)** ^1^H-NMR spectra of methacrylate monomer containing N-halamine compound (ppm, CDCl_3_).

The FT-IR results of PMMA bone cement and antibacterial bone cement materials with different concentrations of the N-halamine compound monomer are shown in Figure 2A. In addition to the characteristic peaks generated by pure PMMA bone cement at 2,952 cm^-1^, 1730 cm^−1^, 1,242 cm^−1^, and 1,154 cm^−1^, the antibacterial bone cement with the N-halamine compound monomer exhibited a distinct peak at 812 cm^-1^, attributed to the stretching vibration of the C-N bond. Furthermore, the original signal peak observed at 1,154 cm^−1^ moved to 1,126 cm^−1^ due to the N-Cl bond induction. With the increase in the concentration of the N-halamine compound monomer, the associated characteristic peak strength of the C-N bond increased, indicating that more monomers were involved in copolymerization.

### 3.2 ^1^H-NMR analysis of methacrylate monomer containing the N-halamine compound

The results of the ^1^H-NMR determination of the methacrylate monomer containing N-halamine compounds are shown in Figure 2B. Notably, the hydrogen on the carbon atom of the olefin exhibited restricted rotation due to the double bond, and when subjected to different deshielding effects, distinct chemical shifts were observed at 6.17 and 5.67 ppm, respectively. The electron cloud of hydrogen on the methyl structure was denser, showed less displacement due to weaker deshielding effects, and was located at 1.95 ppm on the spectrum.

In the structure of 4-methylpiperidinol, the hydrogen within the methine group was subjected to stronger deshielding effects and manifested at 5.17 ppm, whereas the hydrogen in the methylene and methyl groups experienced weaker deshielding effects, corresponding to chemical shifts around 2.13, 1.75, and 1.56 ppm, respectively. The type and quantity of hydrogen in the above-mentioned products corresponded to those in the methacrylate monomer containing the N-halamine compound, indicating that the properties of the products were consistent with the monomer structure.

### 3.3 Determination of antibacterial properties

#### 3.3.1 Contact antibacterial performance test

**FIGURE 3:**
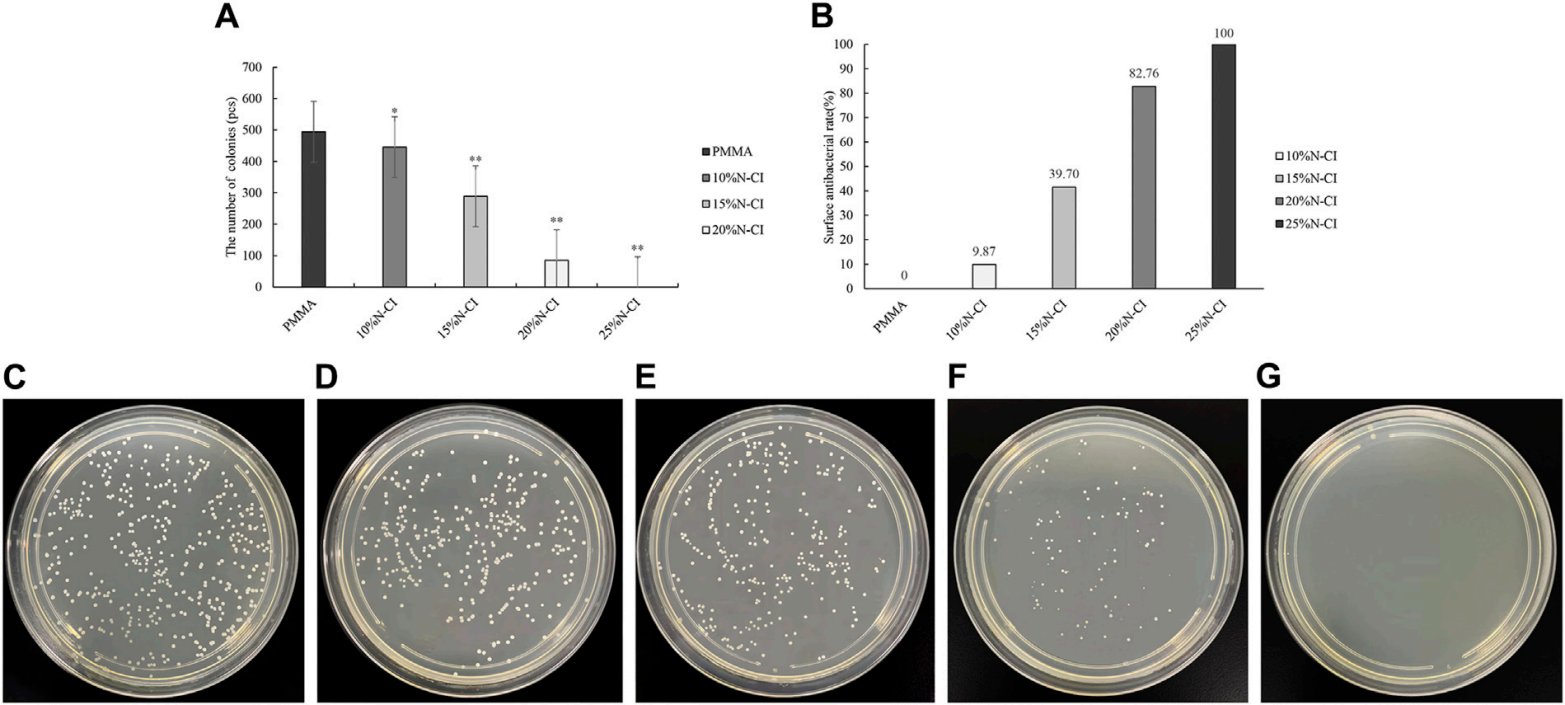
Detection of contact antibacterial performance. **(A)** The number of bacteriostatic colonies of contact bacteriostasis (compared with the PMMA group, * indicates *p* < 0.05, ** indicates *p* < 0.001). **(B)** Contact bacteriostasis rate. **(C–G)** show the colonies of *Staphylococcus aureus* in the dishes of PMMA bone cement and bone cements with 10%, 15%, 20%, and 25% N-halamine compound monomers, respectively. In the dish shown in f, the activity of *Staphylococcus aureus* is reduced. No *Staphylococcus aureus* colonies are formed in the dish shown in **(G)**.

The results of contact antibacterial experiments of the bone cements in each group are shown in Figure 3. Statistical analysis using ANOVA revealed that the number of colonies in the bone cements with 10%, 15%, 20%, and 25% N-halamine compound groups was lower than that in the PMMA group (*p* < 0.05) (Figure 3A). Notably, when the concentration of the N-halamine compound was 15%, the contact antibacterial rate was as high as 39.70% (Figure 3B). As the concentration of the N-halamine compound increased, a gradual decrease in the number of colonies on the surface of bone cement was observed, accompanied by a corresponding increase in the contact bacteriostasis rate (Figures 3C–E). When 25% N-halamine compound was added to the bone cement, the contact bacteriostasis rate reached 100%, with no *S. aureus* colonies detected in the dish (Figures 3F, G).

#### 3.3.2 Detection of releasing-type antibacterial performance tests

**FIGURE 4:**
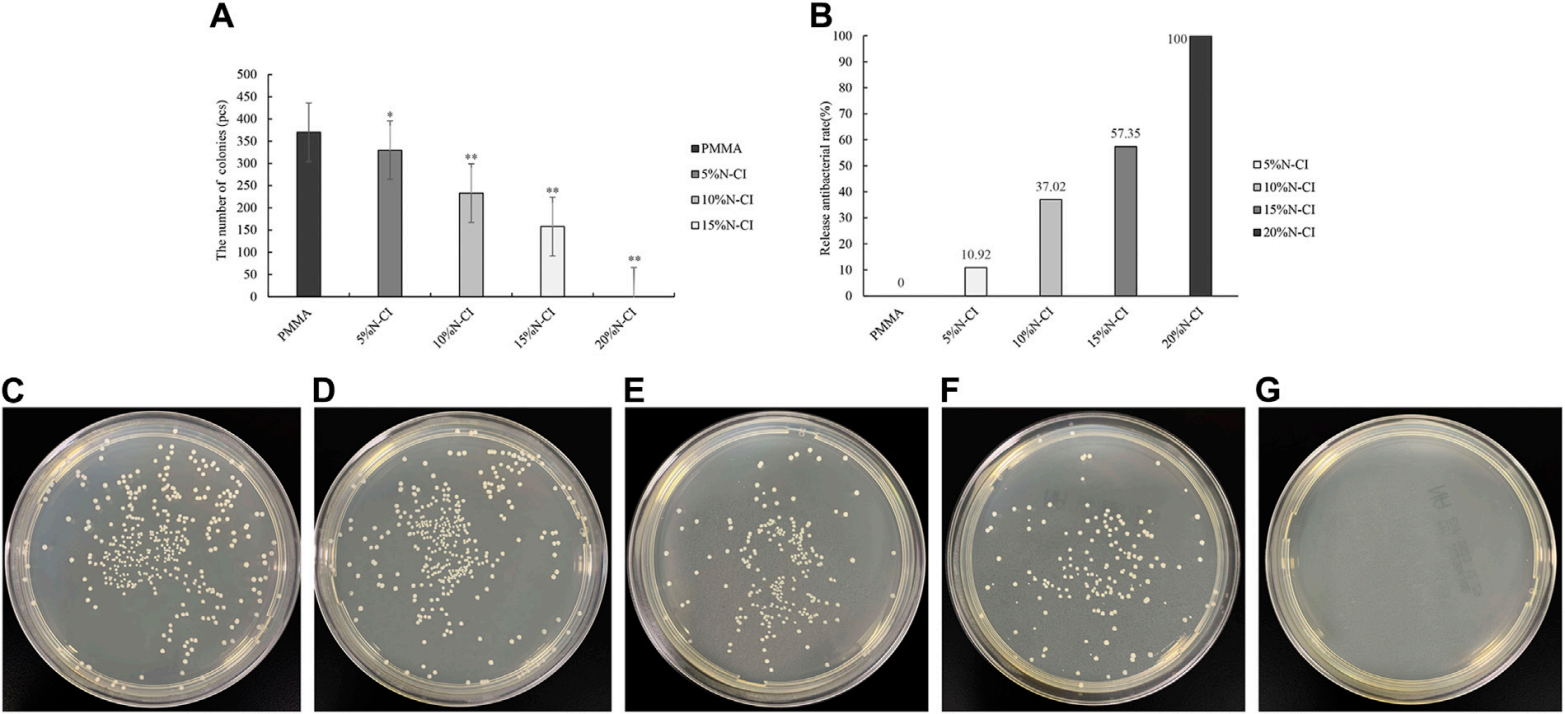
Detection of releasing-type antibacterial performance. **(A)** The number of bacteriostatic colonies of releasing-type bacteriostasis (compared with the PMMA group, * indicates *p* < 0.05, ** indicates *p* < 0.001). **(B)** Releasing-type bacteriostasis rate. **(C–G)** show the colonies of *Staphylococcus aureus* in the dishes of PMMA bone cement and bone cements with 5%, 10%, 15%, and 20% N-halamine compound monomers, respectively. In the dish shown in f, the activity of *Staphylococcus aureus* is reduced. No *Staphylococcus aureus* colonies are formed in the dish shown in **(G)**.

The results of the releasing-type antibacterial test of bone cement in each group are shown in Figure 4. ANOVA results showed that the number of colonies observed in bone cements with 5%, 10%, 15%, and 20% N-halamine compound groups was significantly’ lower than that in the PMMA group (*p* < 0.05) (Figure 4A). When the amount of N-halamine compound was 15%, the releasing-type bacteriostasis rate was significantly increased to 57.35% (Figure 4B). With a gradual increase in the concentration of N-halamine compounds, the number of colonies in the bone cement solution gradually decreased, accompanied by a gradual increase in the releasing-type bacteriostasis rate (Figures 4C–E). Notably, when the concentration of N-halamine compound in the bone cement was 20%, the releasing-type bacteriostasis rate reached 100%, and no *S. aureus* colonies were formed in the dish (Figures 4F, G).

### 3.4 Biocompatibility experiments

#### 3.4.1 *In vitro* cell toxicity experiments

**FIGURE 5:**
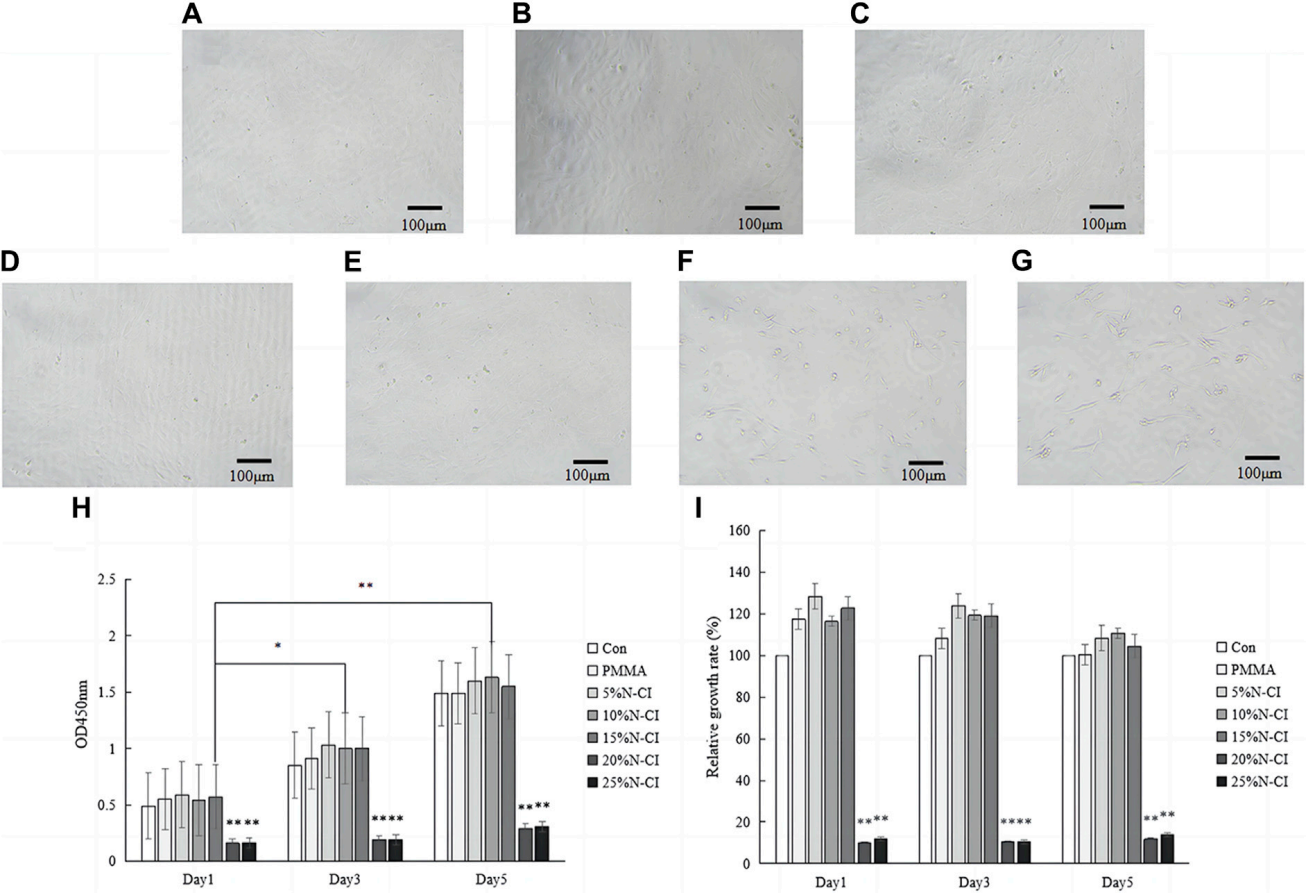
*In vitro* cell proliferation and toxicity experiments. **(A–G)** show the morphology of MC3T3-E1 cells in the control group, PMMA bone cement group, and bone cement groups with 5%, 10%, 15%, 20%, and 25% N-halamine compound monomers, respectively, on day 5 as observed under the microscope. **(H,I)** show the OD values and relative proliferation rates of cells in each group at different time points (when the concentration of N-halamine compound monomer is 15%, the OD values of cells on days 3 and 5 are compared with those on day 1, * indicates *p* < 0.05; the bone cement with 20% and 25% N-halamine compound monomer groups are compared with the blank control group, ** indicates *p* < 0.001).

The morphology of MC3T3-E1 cells was observed under the microscope. On day 5, the morphology of MC3T3-E1 cells in the control group, PMMA bone cement group, and bone cement groups with 5%, 10%, and 15% N-halamine compound monomer was normal, and the cells in the division stage were of irregular spindle or triangular shapes. On day 5, the cells in the bone cement groups with 20% and 25% N-halamine compound monomer were loosely attached, and rounding cells were seen (Figures 5F, G).

The optical density (OD) values of the cultured cells on days 1, 3, and 5 were determined by the CCK-8 method, and the relative proliferation rate of cells was calculated (Figures 5H, I). Repeated measures ANOVA showed that the OD values of cells in the bone cement groups with 5%, 10%, and 15% N-halamine compound monomer increased with time, and the difference was significant (*p* < 0.05). The relative proliferation rate was greater than 100%, and no cytotoxicity was noticed. However, compared with the control group, the OD values of cells and relative proliferation rates in the bone cement groups with 20% and 25% N-halamine compound monomers were significantly reduced (*p* < 0.001), indicating moderate cytotoxicity. Notably, when the concentration of the N-halamine compound monomer reached 15%, the OD value of cells exhibited gradual increments over time (*p* < 0.05), indicating favorable cell proliferation without obvious cytotoxic reactions.

#### 3.4.2 Acute toxicity test in mice

The body weight of all experimental groups of mice increased over the 3 days compared to the first day, indicating a natural growth state, and the differences were statistically significant (*p* < 0.05). The differences in body weight gain at the same time points compared to the saline group were not statistically significant (*p* > 0.05) (Table 2).

**TABLE 2:** Changes in body weight of mice before and after intraperitoneal injection ( $\bar{x}$ ±*s*, g).

Groups	n	Weight change at different time points		
		Day 1	Day 2	Day 3
Normal saline	5	0.56 ± 0.52	0.79 ± 0.46*	0.62 ± 0.50*
PMMA	5	0.61 ± 0.17	0.78 ± 0.17*	0.70 ± 0.09*
5%N-Cl	5	0.60 ± 0.21	0.83 ± 0.18*	0.90 ± 0.29*
10%N-Cl	5	0.23 ± 0.36	0.49 ± 0.37*	0.70 ± 0.42*
15%N-Cl	5	0.56 ± 0.56	0.74 ± 0.66*	0.56 ± 0.80
20%N-Cl	5	0.59 ± 0.54	0.74 ± 0.65*	0.74 ± 0.74*
25%N-Cl	5	0.31 ± 0.52	0.44 ± 0.58*	0.69 ± 0.64*
F_intergroup/time/interaction_ *P*_intergroup/time/interaction_	0.278/21.623/2.374 0.943/<0.001/0.021			

Note: Fintergroup represents the overall comparison of mouse body weight changes at the same time points between the PMMA, bone cement group and the groups with 5%, 10%, 15%, 20%, and 25% N-halamine compound monomer added bone cement, respectively, compared with the saline group (blank control group), and the comparison with the saline group at the same time points showed *p* > 0.05.

Ftime represents the overall comparison of mouse body weight changes at different time points within each group compared with the body weight change at day 1, and compared with the body weight change at day 1, * indicates *p* < 0.05.

**FIGURE 6:**
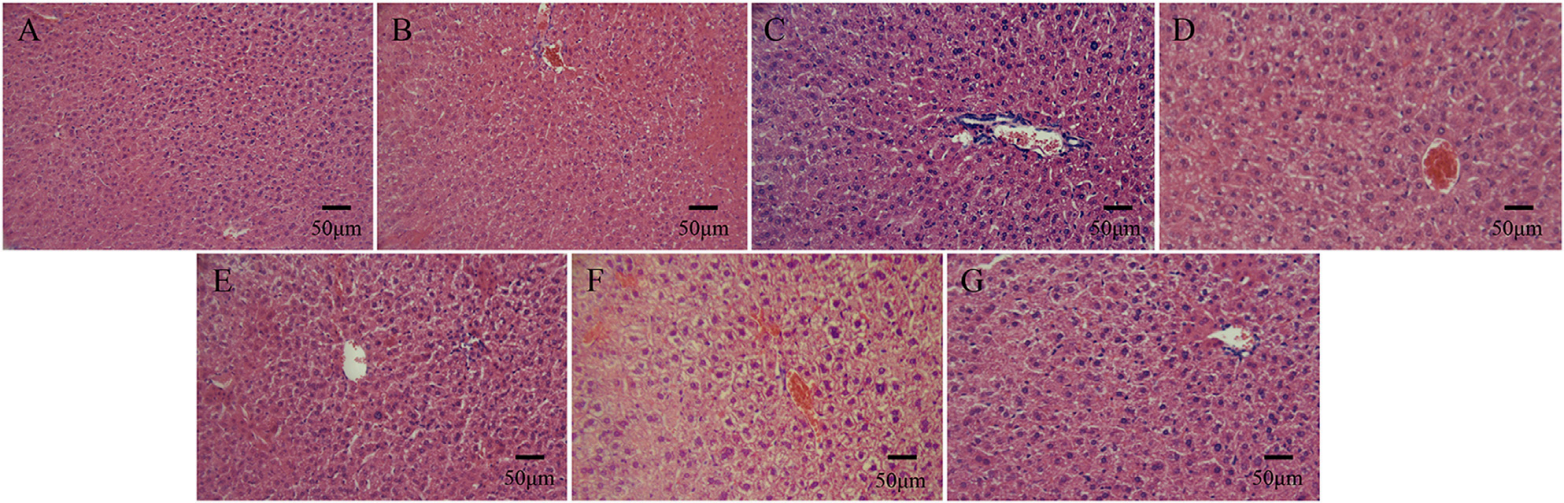
Histological staining images of mice livers on day 3 after the intraperitoneal injection, as observed under a light microscope. **(A–G)** show histological staining images of mice livers on day 3 after the injection in the control group, PMMA bone cement group, and bone cement groups with 5%, 10%, 15%, 20%, and 25% N-halamine compound monomers (hematoxylin-eosin staining).

On days 1, 2, and 3 after the intraperitoneal injection, none of the mice exhibited symptoms such as dyspnea, decreased activity, tremors, diarrhea, and the like, and the weight of all the experimental mice showed a natural increasing trend. Compared with the normal saline group, the morphological structure of the cells in the hepatic lobular area of mice injected with bone cement extracts of different concentrations of N-halamine compound monomers was normal, and there was no interstitial congestion or obvious inflammatory cell infiltration. There was no significant swelling or loosening observed in the hepatocytes of any of the mice (Figure 6).

**FIGURE 7:**
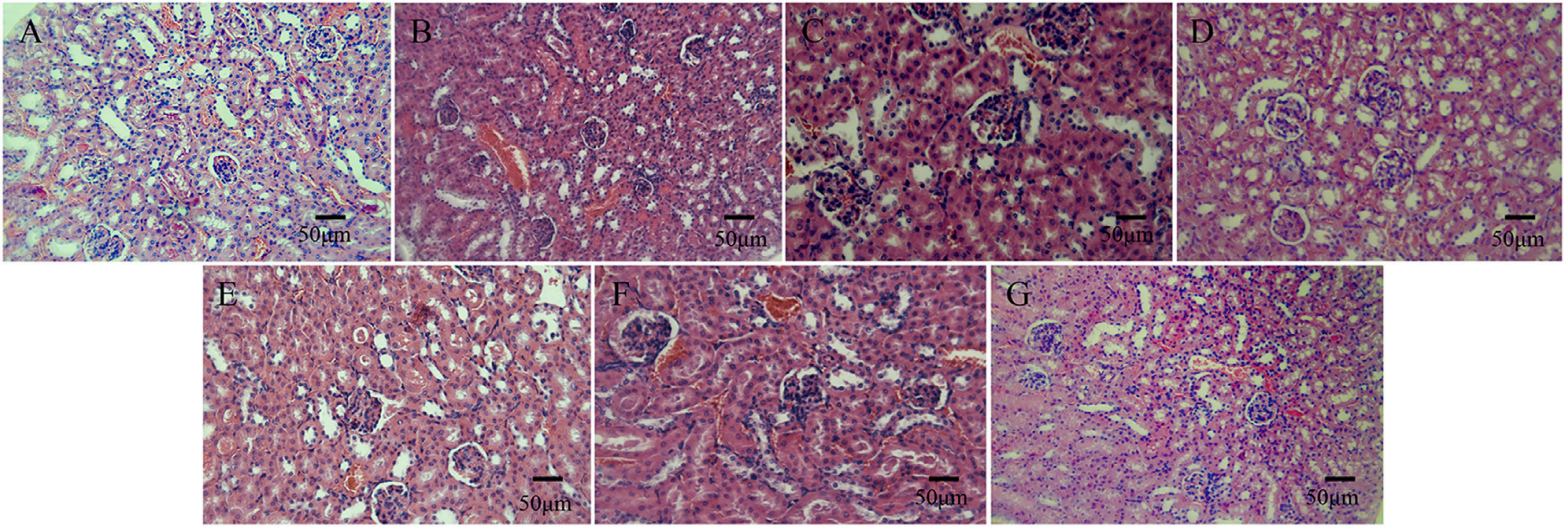
Histological staining images of mice kidneys on day 3 after the intraperitoneal injection, as observed under a light microscope. **(A–G)** show the histological staining images of mice kidneys on day 3 after the intraperitoneal injection in the control group, PMMA bone cement group, and bone cement groups with 5%, 10%, 15%, 20%, and 25% N-halamine compound monomers (hematoxylin-eosin staining).

In the histological observation of the kidneys of the mice, in comparison with the normal saline group, the morphological structure of the renal cortex and renal medullary cells of mice injected with bone cement extracts of different concentrations of N-halamine compound monomers was normal, and there was no interstitial inflammatory cell infiltration. No obvious abnormalities were found in the glomerular and tubular morphology, and there was no edema, necrosis, or detachment in the renal tubular epithelial cells (Figure 7).

### 3.5 Mechanical properties

With the increase in the addition of the N-halamine compound monomer, both the compressive strength and the elastic modulus of the novel antibacterial bone cement gradually decreased. When the addition of N-halamine compound monomers reached 15%, the compressive strength and elastic modulus were 34.00 and 1,100.62 MPa, respectively, lower than those of the PMMA group (Supplementary Figure S1).

## 4 Discussion

To address the many challenges associated with ALBC, a new type of antibacterial bone cement was developed by the researchers in this study. This method involved using the covalent linkage strategy of antibacterial groups, wherein chemical groups possessing antibacterial properties are covalently linked with the MMA monomer side chain of the bone cement, resulting in the development of PMMA bone cement containing a new type of antibacterial monomer, known as non-leaching antibacterial bone cement (NLBC).

Chu (Chu et al., 2022) developed a bio-based bone cement utilizing a novel modified methyl methacrylate-5-nitrofurfuryl methacrylate (p(NFMA-co-MMA)) composition and found that incorporating a specific proportion of the p(NFMA-co-MMA) monomer significantly reduced the polymerization temperature and prolonged the working time of traditional PMMA bone cement. In addition, when the concentration of the p(NFMA-co-MMA) monomer was 10%, the contact antibacterial efficiency of the new bone cement against *Escherichia coli* and *S. aureus* was 97.5% and 99.6%, respectively, and it showed good biocompatibility both *in vitro* and *in vivo*.

Zhu et al. (2017) synthesized a new type of antibacterial bone cement containing the methacrylate monomer BTTMA with a benzothiazole structure and found that, with the progressive addition of BTTM, the compressive strength and elastic modulus of the new bone cement were significantly higher than those of the control group. When the BTTM concentration was 15%, it showed 54% of antibacterial effects on *S. aureus* without inducing hemolysis, thus demonstrating good *in vitro* biocompatibility.

In their study, Wu et al. (2021) reported that a porous N-halamine polymer coating, Ti-PAA-NCl, applied to the surface of titanium showed a 64% inhibition rate of *S. aureus* and a 42% inhibition rate of *Porphyromonas gingivalis*. They demonstrated that the coating exerted its antibacterial activity through two mechanisms: contact killing and releasing-type killing. Another notable finding was that the *S. aureus* and *P. gingivalis* biofilms that adhered to the surface of the novel coating were damaged, indicating that the Ti-PAA-NCl material was not only effective in killing pathogenic bacteria but also in preventing the formation of bacterial biofilms.

Building upon previous studies on N-halamine compounds and considering the exothermic polymerization of PMMA bone cement material, an amine N-halamine compound monomer was prepared in this study. This monomer is designed to be more stable than amide N-halamine and imide N-halamine (Wu et al., 2021). Furthermore, the FT-IR spectra of PMMA bone cement materials containing different concentrations of N-halamine compound monomers were analyzed. The results showed that the signal peak observed at 812 cm^−1^ represented a characteristic peak generated by the C-N bond stretching vibration in the antibacterial cement material containing the N-halamine compound. Moreover, the induction of the N-Cl bond led to the displacement of the characteristic peak observed in the original PMMA spectrum. In addition, ^1^H-NMR spectra analysis of methacrylate monomers containing the N-halamine compound showed that the type and quantity of hydrogen in the product corresponded to those found in methacrylate monomers containing N-halamine compounds, thus indicating consistency between the properties of the product and the monomer structure.

The antibacterial mechanisms of N-halamine can be divided into contact bacteriostasis, releasing-type bactericidal effects, and transferring bacteriostasis. (Sun et al., 1994; Engel et al., 2012; Laingam et al., 2012; Li et al., 2012; Sun et al., 2012; Bastarrachea et al., 2013; Yu et al., 2013; Farah et al., 2015; Natan et al., 2015; Farah et al., 2016). In the mechanism of contact bacteriostasis, active halogens are directly transferred from the N-halamine compound to bacterial surface receptors, while the halogens remain bound to the N-X bond. Conversely, in the releasing-type bactericidal effect, the active halogens initially dissociate from the N-X bond and subsequently migrate into the solution during the releasing-type bactericidal process or into the media component during the transferring bactericidal process.

For analyzing the antibacterial properties of the new N-halamine bone cement, we conducted separate contact bacteriostasis and releasing-type bacteriostasis experiments using *S. aureus*, a commonly seen pathogen in orthopedic implant infections. The results showed that the novel N-halamine compound methacrylate copolymer cement operated through a combined mechanism involving both contact bacteriostasis and releasing-type bacteriostasis. This dual-action mechanism offered enhanced antibacterial protection for the implant.

In addition, owing to the relatively stable structure of the N-chloramine designed and synthesized in this study, its contact bacteriostatic ability and releasing-type bactericidal ability were comparable, with the releasing-type bactericidal ability slightly better than contact bacteriostatic ability. In the releasing-type bactericidal experiment, the initial releasing-type bacteriostasis performance was observed when the content of the N-halamine compound in the bone cement was 5%. In the contact bacteriostasis experiment, the initial contact bacteriostasis performance was seen only when the concentration of the N-halamine compound in the bone cement was 10%. However, when the concentration of the N-halamine compound increased from 15% to 20%, the rate of releasing-type bacteriostasis increased sharply from 57.35% to 100%. This may be attributed to the increased dissociation of the active halogen Cl+ from the N-X bond as the addition of the N-halamine compound increased, resulting in a “sudden releasing effect”. Consequently, this led to the death of a substantial number of *S. aureus* in the bacterial solution and a sharp increase in the rate of releasing-type bacteriostasis.

Irrespective of whether it is contact bacteriostasis or the releasing-type bactericidal effect, active halogen serves as the main component of N-halamine responsible for exerting its bactericidal effects. In the case of N-chloramine, the initial target of active Cl^+^ is to chlorinate the protein matrix on the outside of the bacteria, and the active Cl^+^ forms a covering around the bacteria. This layer helps it penetrate into the bacterial cells, further oxidizing many key cellular components with thiol and thioether structures and denaturing the proteins through trans-chlorine interactions, ultimately causing the bacteria to lose their activity and die (Gottardi et al., 2013; De Silva et al., 2015; Dong et al., 2017). Due to the non-specific interaction between N-halamine and bacteria-important proteins, N-halamine compounds are thought to have broad-spectrum antibacterial susceptibility. Furthermore, even if N-halamine copolymer bone cement persists within local tissues for an extended period of time, it is unlikely to induce bacterial resistance.

Good biocompatibility is the most fundamental property of biomedical materials. When they performed CCK-8 detection on porous N-halamine polymer coating (Ti-PAA-NCl), (Wu et al., 2021) found that there was no significant difference in the growth and proliferation of osteocyte precursor cells (MC3T3-E1) between the Ti-PAA-NCl group and the control group. Additionally, the fluorescence staining results showed that Ti-PAA-NCl did not affect cell proliferation or adhesion. Similarly, (Umair et al., 2015) fabricated N-halamine-based surgical bactericidal sutures through the deposition of N-halamine-4-piperidinol polymer on polyglycolide sutures. They found that the N-halamine sutures effectively inactivated the experimental strain in a short period of time when the chlorine load was 0.22%, and these sutures also showed good biocompatibility in the *in vitro* hemolysis experiment and the cytocompatibility test.

In this study, the biocompatibility of the novel antibacterial bone cement was evaluated using the CCK-8 method and testing for acute systemic toxicity in mice. In the experimental study of the proliferation and toxicity of osteoblast precursor cells (MC3T3-E1), it was found that, compared to the untreated blank group with cell culture medium, the cell morphology of the PMMA bone cement group and the bone cement groups with 5%, 10%, and 15% N-halamine compound monomers was normal at each time point. The cells in the division stage exhibited irregular spindle or triangular shapes, indicating a normal proliferation state, with a relative proliferation rate greater than 100%, and no cytotoxicity was detected. However, on day 5 of cell culture, the cells in the bone cement groups with 20% and 25% N-halamine compound monomers were poorly attached, indicating moderate cytotoxicity. This outcome may be related to the “sudden releasing effect” of active Cl^+^. With an increasing concentration of the N-halamine compound, a large amount of active halogen Cl + dissociated from the N-X bond, resulting in the non-specific killing of MC3T3-E1 cells.

In the acute systemic toxicity experiment involving mice, the general condition of mice after injection of different concentrations of N-halamine compound monomers and bone cement extracts was observed and compared to the normal saline group. It was found that no mice showed symptoms such as dyspnea, decreased activity, tremors, diarrhea, or other symptoms at any time point throughout the period of observation. Moreover, the weight of the mice showed a natural increasing trend. Histological observation of the liver and kidney of the mice showed that the hepatic and renal cells of all the experimental mice displayed good morphology, and there was no interstitial inflammatory cell infiltration. These results collectively indicate that the new N-halamine compound monomer copolymer bone cement exhibited favorable biocompatibility properties *in vivo*.

## 5 Conclusion

In this study, a novel antibacterial PMMA bone cement containing a methacrylate monomer with an N-halamine compound was designed and synthesized, and this demonstrated good antibacterial efficacy against *S. aureus* via both contact bacteriostasis and releasing-type bactericidal effect mechanisms. Notably, the bone cement with a 15% N-halamine compound showed good biocompatibility both *in vitro* and *in vivo* while exerting strong antibacterial effects on *S. aureus*. In conclusion, the antibacterial PMMA bone cement containing methacrylate monomer with a 15% N-halamine compound had the best overall performance in this study, suggesting its potential as a promising candidate for the development of novel antibacterial orthopedic implant materials in the future.

## Data Availability

The original contributions presented in the study are included in the article/Supplementary Material, further inquiries can be directed to the corresponding author.

## Supplementary material

SUPPLEMENTARY FIGURE S1 Compressive strength and elastic modulus results of bone cement. **(A)** Compressive strength, **(B)** Elastic modulus (compared with the PMMA group, ** indicates *p* < 0.001).

## Abbreviations

ALBC, (antibiotic-loaded bone cement); PJI, (periprosthetic joint infection); PMMA, (polymethyl methacrylate); MMA, (methyl methacrylate); NLBC, (non-leaching bone cement); BPO, (benzoyl peroxide); DMPT, (N,N-Dimethyl-p-toluidine); EDCI, (1-Ethyl-3- (3′-dimethylaminopropyl)-carbodiimide Hydrochloride); DMAP, (4-dimethylaminopyridine); DCM, (dichloromethane); CCK-8, (Cell Counting Kit-8); OD, (optical density); RGR, (relative growth rate).

## References

[B1] Al Thaher Y. Perni S. Prokopovich P. (2017). Nano-carrier based drug delivery systems for sustained antimicrobial agent release from orthopaedic cementous material. Adv. Colloid Interface Sci. 249, 234–247. 10.1016/j.cis.2017.04.017 28477865

[B2] Bai R. Zhang Q. Li L. Li P. Wang Y. J. Simalou O. (2016). N-halamine-containing electrospun fibers kill bacteria via a contact/release co-determined antibacterial pathway. ACS Appl. Mat. Interfaces 8, 31530–31540. 10.1021/acsami.6b08431 27808500

[B3] Balato G. Roscetto E. Vollaro A. Galasso O. Gasparini G. Ascione T. (2019). Bacterial biofilm formation is variably inhibited by different formulations of antibiotic-loaded bone cement *in vitro* . Knee Surg. Sports Tr. A 27, 1943–1952. 10.1007/s00167-018-5230-x 30370437

[B4] Bastarrachea L. J. Peleg M. McLandsborough L. A. Goddard J. M. (2013). Inactivation of Listeria monocytogenes on a polyethylene surface modified by layer-by-layer deposition of the antimicrobial N-halamine. J. Food Eng. 117, 52–58. 10.1016/j.jfoodeng.2013.02.004

[B5] Bhattacharjee B. Ghost S. Patra D. Haldar J. (2022). Advancements in release‐active antimicrobial biomaterials: a journey from release to relief. Wiley Interdiscip. Rev. Nanomed. Nanobiotechnol 14 (1), e1745. 10.1002/wnan.1745 34374498

[B6] Boelch S. P. Jordan M. C. Arnholdt J. Rudert M. Luedemann M. Steinert A. F. (2017). Loading with vancomycin does not decrease gentamicin elution in gentamicin premixed bone cement. J. Mater Sci-Mater M. 28, 104–107. 10.1007/s10856-017-5915-6 28534287

[B7] Chu J. J. Li C. Guo J. Xu Y. Fu Y. (2022). Preparation of new bio-based antibacterial acrylic bone cement via modification with a biofunctional monomer of nitrofurfuryl methacrylate. Polym. Chem-UK 13 (32), 4675–4683. 10.1039/d2py00235c

[B8] Deb S. Doiron R. Disilivo L. Punyani S. Singh H. (2008). PMMA bone cement containing a quaternary amine comonomer with potential antibacterial properties. J. Biomed. Mater Res. B Appl. Biomater. 85 (1), 130–139. 10.1002/jbm.b.30925 17806110

[B9] De Silva M. Ning C. Ghanbar S. Zhanel G. Logsetty S. Liu S. (2015). Evidence that a novel quaternary compound and its organic N-chloramine derivative do not select for resistant mutants of *Pseudomonas aeruginosa* . J. Hosp. Infect. 91, 53–58. 10.1016/j.jhin.2015.05.009 26122622

[B10] Dong A. Wang Y. J. Gao Y. Gao T. Gao G. (2017). Chemical insights into antibacterial N-halamines. Chem. Rev. 117, 4806–4862. 10.1021/acs.chemrev.6b00687 28252944

[B11] Engel Y. Schiffman J. D. Goddard J. M. Rotello V. M. (2012). Nanomanufacturing of biomaterials. Mat. Today 15, 478–485. 10.1016/s1369-7021(12)70217-1

[B12] Farah S. Aviv O. DaifKunduru M. K. R. Laout N. Ratner S. Beyth N. (2016). N-Bromo-Hydantoin grafted polystyrene beads: synthesis and nano-micro beads characteristics for achieving controlled release of active oxidative bromine and extended microbial inactivation efficiency. J. Polym. Sci. Part A Polym. Chem. 54, 596–610. 10.1002/pola.27894

[B13] Farah S. Aviv O. Laout N. Ratner S. Domb A. J. (2015). Antimicrobial N-brominated hydantoin and uracil grafted polystyrene beads. J. Control. Release 216, 18–29. 10.1016/j.jconrel.2015.07.013 26220618

[B14] Ghimire A. Song J. (2021). Anti-periprosthetic infection strategies: from implant surface topographical engineering to smart drug-releasing coatings. ACS Appl. Mater interfaces 13 (18), 20921–20937. 10.1021/acsami.1c01389 33914499 PMC8130912

[B15] Gottardi W. Debabov D. Nagl M. (2013). N-chloramines, a promising class of well-tolerated topical anti-infectives. Antimicrob. Agents Chemother. 57, 1107–1114. 10.1128/aac.02132-12 23295936 PMC3591902

[B16] Jiao Y. Niu L. Ma S. Li J. Tay F. R. Chen J. h. (2017). Quaternary ammonium-based biomedical materials: state-of-the-art, toxicological aspects and antimicrobial resistance. Prog. Polym. Sci. 71, 53–90. 10.1016/j.progpolymsci.2017.03.001 32287485 PMC7111226

[B17] Laingam S. Froscio S. M. Bull R. J. Humpage A. R. (2012). *In vitro* toxicity and genotoxicity assessment of disinfection by-products, organic N-chloramines. Environ. Mol. Mutage 53, 83–93. 10.1002/em.20684 22403827

[B18] Li L. Pu T. Zhanel G. Zhao N. Ens W. Liu S. (2012). New biocide with both N-chloramine and quaternary ammonium moieties exerts enhanced bactericidal activity. Adv. Healthc. Mater 1, 609–620. 10.1002/adhm.201200018 23184796

[B19] Li T. (2014) Study on bioactivity and antibacterial activity of PMMA bone cement [D]. Peking Union Medical College.

[B20] Liu C. Shan H. Chen X. Si Y. Yin X. Yu J. (2018). Novel inorganic-based N-halamine nanofibrous membranes as highly effective antibacterial agent for water disinfection. ACS Appl. Mat. Interfaces 10, 44209–44215. 10.1021/acsami.8b18322 30525383

[B21] Luo J. Porteous N. Sun Y. (2011). Rechargeable biofilm-controlling tubing materials for use in dental unit water lines. ACS Appl. Mat. Interfaces 3, 2895–2903. 10.1021/am200576q PMC3161146 21721534

[B22] Ma W. Li L. Lin X. Wang Y. Ren X. Huang T. S. (2019a). Novel ZnO/N-halamine-mediated multifunctional dressings as quick antibacterial agent for biomedical applications. ACS Appl. Mat. Interfaces 11, 31411–31420. 10.1021/acsami.9b10857 31373785

[B23] Ma Y. Li J. Si Y. Huang K. Nitin N. Sun G. (2019b). Rechargeable antibacterial N-halamine films with antifouling function for food packaging applications. ACS Appl. Mat. Interfaces 11, 17814–17822. 10.1021/acsami.9b03464 31022343

[B24] Miao X. Yang S. Zhu J. Gong Z. Wu D. Hong J. (2023). Bioactive mineralized small intestinal submucosa acellular matrix/PMMA bone cement for vertebral bone regeneration. Regen. Biomater. 10, rbad040. 10.1093/rb/rbad040 37250976 PMC10224805

[B25] Natan M. Gutman O. Lavi R. Margel S. Banin E. (2015). Killing mechanism of stable N-halamine cross-linked polymethacrylamide nanoparticles that selectively target bacteria. ACS Nano 9, 1175–1188. 10.1021/nn507168x 25602279

[B26] Nau C. Seebach C. Trumm A. Schaible A. Kontradowitz K. Meier S. (2016). Alteration of Masquelet's induced membrane characteristics by different kinds of antibiotic enriched bone cement in a critical size defect model in the rat's femur. Injury 47 (2), 325–334. 10.1016/j.injury.2015.10.079 26652225

[B27] Schnieders J. Gbureck U. Vorndran E. Schossig M. Kissel T. (2011). The effect of porosity on drug release kinetics from vancomycin microsphere/calcium phosphate cement composites. J. Biomed. Mat. Res.B 99 (2), 391–398. 10.1002/jbm.b.31910 21948487

[B28] State Administration for Market Regulation of China (2022) Biological evaluation of medical devices:GB/T 16886-2021[S]. Beijing: Standards Press of China, 6–11.

[B29] Sun G. Wheatley W. B. Worley S. D. (1994). A new cyclic N-halamine biocidal polymer. Ind. Eng. Chem. Res. 33, 168–170. 10.1021/ie00025a022

[B30] Sun X. Cao Z. Porteous N. Sun Y. (2012). An N-Halamine-Based rechargeable antimicrobial and biofilm controlling polyurethane. Acta Biomater. 8, 1498–1506. 10.1016/j.actbio.2011.12.027 22244984 PMC3289718

[B31] Umair M. M. Jiang Z. Safdar W. Xie Z. Ren X. (2015). N-Halamine-Modified polyglycolide (PGA) multifilament as a potential bactericidal surgical suture: *in vitro* study. J. Appl. Polym. Sci. 132, 42483–42491. 10.1002/app.42483

[B32] Vasilev K. Cook J. Griesser H. J. (2009). Antibacterial surfaces for biomedical devices. Expert Rev. Med. Devices 6 (5), 553–567. 10.1586/erd.09.36 19751126

[B33] Wu S. Xu J. Zou L. Luo S. Yao R. Zheng B. (2021). Long-lasting renewable antibacterial porous polymeric coatings enable titanium biomaterials to prevent and treat peri-implant infection. Nat. Commun. 12 (1), 3303. 10.1038/s41467-021-23069-0 34083518 PMC8175680

[B34] Yu H. Zhang X. Zhang Y. Liu J. Zhang H. (2013). Development of a hydrophilic PES ultrafiltration membrane containing SiO2@N-Halamine nanoparticles with both organic antifouling and antibacterial properties. Desalination 326, 69–76. 10.1016/j.desal.2013.07.018

[B35] Zhu W. Liu F. He J. (2017). Synthesis of imidazolium-containing mono-methacrylates as polymerizable antibacterial agents for acrylic bone cements. J. Mech. Behav. Biomed. Mater 74, 176–182. 10.1016/j.jmbbm.2017.06.003 28601760

