# Supplementary figures and images for: Preparation and efficacy of antibacterial methacrylate monomer-based polymethyl methacrylate bone cement containing N-halamine compounds
Source: Front Bioeng Biotechnol. 2024 May 28;12:1414005. doi: 10.3389/fbioe.2024.1414005 (PMC11165117; doi:10.3389/fbioe.2024.1414005)

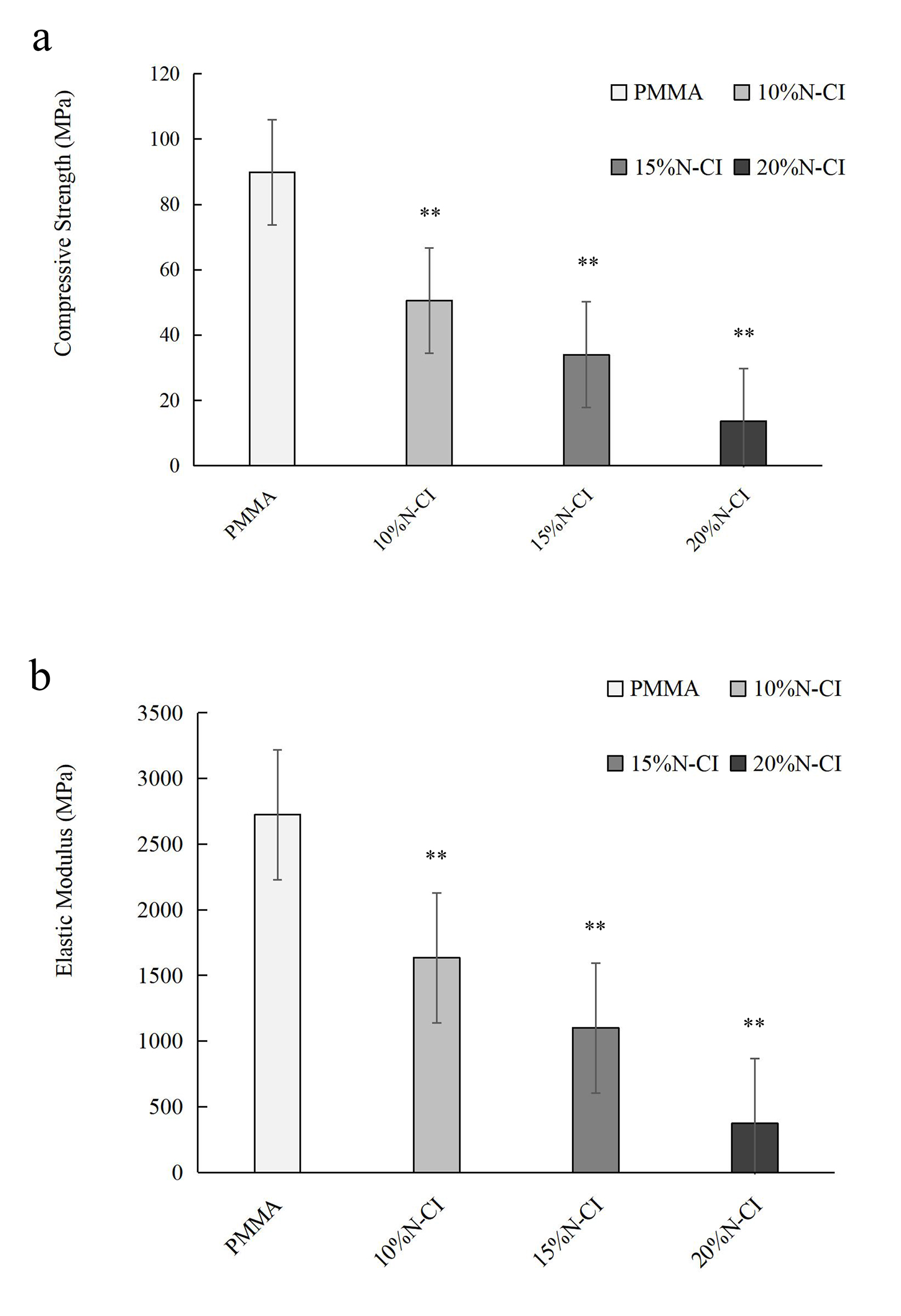

Supplement: Supplementary file 1 [file Image1.jpg]
